# Combining Object Detection, Super-Resolution GANs and Transformers to Facilitate Tick Identification Workflow from Crowdsourced Images on the eTick Platform

**DOI:** 10.3390/insects16080813

**Published:** 2025-08-06

**Authors:** Étienne Clabaut, Jérémie Bouffard, Jade Savage

**Affiliations:** 1 Département de Géomatique Appliquée, Université de Sherbrooke, Sherbrooke, QC J1K 2R1, Canada; 2 Biological Sciences, Bishop’s University, 2600 College Street, Sherbrooke, QC J1M 1Z7, Canada

**Keywords:** deep learning, super resolution, *Ixodes scapularis*, *Amblyomma americanum*, ixodida, tick surveillance, citizen science

## Abstract

Ongoing changes in the distribution and abundance of several tick species of medical relevance in Canada have prompted the development of the eTick platform—an image-based crowd-sourcing public surveillance tool for Canada enabling rapid tick species identification by trained personnel, and public health guidance based on tick species and province of residence of the submitter. Considering that more than 100,000 images from over 73,500 identified records representing 25 tick species have been submitted to eTick since the public launch in 2018, a partial automation of the image processing workflow could save substantial human resources, especially as submission numbers have been steadily increasing since 2021. In this study, we evaluate an end-to-end artificial intelligence (AI) pipeline to support tick identification from eTick user-submitted images, characterized by heterogeneous quality and uncontrolled acquisition conditions. Our framework integrates (i) tick localization using a fine-tuned YOLOv7 object detection model, (ii) resolution enhancement of cropped images via super-resolution Generative Adversarial Networks (RealESRGAN and SwinIR), and (iii) image classification using deep convolutional (ResNet-50) and transformer-based (ViT) architectures across three datasets (12, 6, and 3 classes) of decreasing granularities in terms of taxonomic resolution, tick life stage, and specimen viewing angle. ViT consistently outperformed ResNet-50, especially in complex classification settings. The configuration yielding the best performance—relying on object detection without incorporating super-resolution—achieved a macro-averaged F1-score exceeding 86% in the 3-class model (*Dermacentor* sp., other species, bad images), with minimal critical misclassifications (0.7% of “other species” misclassified as *Dermacentor*). Given that *Dermacentor* ticks represent more than 60% of tick volume submitted on the eTick platform, the integration of a low granularity model in the processing workflow could save significant time while maintaining very high standards of identification accuracy. Our findings highlight the potential of combining modern AI methods to facilitate efficient and accurate tick image processing in community science platforms, while emphasizing the need to adapt model complexity and class resolution to task-specific constraints.

## Simple Summary

Canada’s eTick platform now receives over 30,000 tick photographs from the public every year, increasing the strain placed on the small team of trained personnel who identify each submission. To ease this burden, we built a three-stage system that processes and sorts images into broad categories. Stage 1—Locate the tick. An object-detection model finds and crops the tick in each photo, succeeding 99.5% of the time. Stage 2—Sharpen the view. The cropped region is passed through a super-resolution network that improves the image of the tick. Stage 3—Photo sorting. A vision transformer places the enhanced cropped images into one of three useful groups: (1) *Dermacentor* ticks, (2) other ticks, or (3) unusable or poor-quality images. Across the test set the system selects the right group in 86% of cases and very rarely makes a serious mistake: fewer than one image in 100 displaying a non-Dermacentor tick was mislabelled as *Dermacentor*. Given that *Dermacentor* ticks represent about 60% of submissions on eTick, this system should automatically filter about two-thirds of photos (*Dermacentor*) to a low-priority queue and request new pictures when pictures are unusable. This will allow the eTick team to focus on challenging and/or high medical relevance submissions while still giving timely feedback to contributors.

## 1. Introduction

Several studies have documented rapid shifts in the abundance and geographic distribution of several tick species across North America, accompanied by a rising incidence of tick-borne diseases affecting both humans and animals [1,2,3,4]. In Canada, the blacklegged tick (*Ixodes scapularis*) and the western blacklegged tick (*Ixodes pacificus*) are the primary vectors for the pathogens responsible for Lyme disease and human granulocytic anaplasmosis [1,5]. In addition to these, a variety of other bacteria, protozoa, and viruses are transmitted by one or more of the approximately 35 tick species established in the country [6]. The potential establishment of non-native species of medical and veterinary concern such as the lone star tick (*Amblyomma americanum*) and the longhorned tick (*Haemaphysalis longicornis*) also poses a significant public health risk [4,7].

Over the past several decades, numerous initiatives have been launched in Canada to support both passive and active surveillance of ticks and tick-borne diseases [5,8,9]. Until recently, specimen-based passive acarological surveillance, where ticks are mailed to diagnostic laboratories for identification, was the only available approach. While this procedure is effective for collecting specimens for pathogen testing, it results in significant delays in identification for the submitter (when results are communicated at all). Moreover, as tick populations have steadily increased across many provinces, this method has become increasingly unsustainable due to the growing volume of submissions and associated resource demands in most provinces.

In response to the limitations of traditional specimen-based surveillance, recent technological advancements have enabled the development of innovative, image-based crowd-sourcing platforms for monitoring wildlife such as iNaturalist [10], eButterfly [11], and eBird [12]. In 2014, a collaboration between Bishop’s University (J. Savage), the Public Health Agency of Canada, and the Laboratoire de Santé Public du Quebec led to the creation of such a tool for ticks, the eTick platform [13,14]. The main distinctions between eTick and iNaturalist (the only other public web platform with tick records across Canada) are that on eTick: (1) all submissions are identified by trained personnel, (2) users receive messages of medical and veterinary relevance tailored to the species encountered and their province of residence within 1–2 business days, and (3) additional surveillance data such as host type, age and sex of the host (if human), as well as recent travel history are collected. To ensure the active collaboration of regional health authorities in the drafting and updating of species identification messages, the platform’s coverage was gradually expanded beyond the province of Quebec starting in 2019, a period when several provinces had already begun to scale back or eliminate their tick surveillance programs.

Between 1 January 2018 and 1 June 2025, eTick received more than 100,000 submissions, resulting in over 73,500 identifiable public records belonging to 25 tick species. In comparison for the same period, the iNaturalist database holds 5830 research-grade records belonging to 14 species. While such a strong public response is a testament to the success of the eTick platform, the large volume of submissions now calls for the development and implementation of new technological tools to streamline and facilitate the eTick identification workflow. To do so, we turned to artificial intelligence (AI) as a means of improving the quality of images submitted to the platform, and to develop algorithms that will assist eTick personnel with the tick identification workflow.

**Figure 1:**
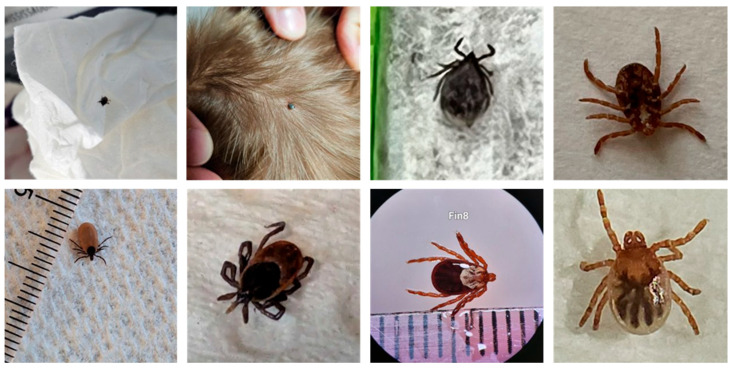
Original images submitted to the eTick platform illustrating the range of picture quality in terms of specimen position, space occupied by the specimen, lighting and resolution.

Artificial intelligence has been used increasingly often in recent years to identify a wide range of plants [15,16] and animals [17,18], including ticks [19,20,21,22]. With the exception of [19], the works involving ticks used deep learning models on “artificially” prepared datasets. This means that pictures were taken under laboratory conditions (standardized specimen positioning and illumination and dedicated photographic equipment) that do not reflect the actual quality expected from untrained users sharing pictures on a public platform. A model trained on such datasets would obviously perform poorly when presented with low-quality images taken from different angles. Such images could be considered as a shift in the data distribution (i.e., an unrepresentative sample), a well-known problem among machine learning specialists [23]. Most pictures submitted on the eTick platform and other similar image-based monitoring programs are taken by mobile phone and many are of poor quality. In many cases, the quality is so poor that identification is not possible even for human experts. Any AI tool developed to improve the sorting of tick images on the eTick platform therefore needs to account for high levels of variability in the quality of original images submitted by users (see Figure 1).

To the best of our knowledge, the published study closest to the work presented here is from [19]. The authors used a “user-generated dataset of more than 12,000 images”. Different architectures were tried such as Inception-V3, ResNet-101, DenseNet-121 and DenseNet-201. It is worth noting that such architectures do not represent the state of the art in the computer vision domain. New architecture approaches that outperform those employed by [19] such as those using transformers like ViT (Vision Transformer; a convolution-free architecture that uses attention mechanisms to model relationships between image patches) have since become available [24]. Moreover, Ref. [19] “cropped (images) to 224 × 224 × 3 input format”. This manual task in the pre-processing of the data is not compatible with an end-to-end automatic identification process. Finally, the authors emphasize that the accuracy of the prediction is proportional to the relative tick size in the image. The bigger the relative size, the higher the accuracy. Fortunately, new technology such as Generative Adversarial Networks (GANs) could be used to mediate this issue by achieving a higher relative size of the tick in the image.

This paper aims to explore how recent developments in AI technology may be applied to community generated image databases to be integrated in the identification workflow of community science programs like eTick. More specifically, we used recent developments in AI technology to (i) automatically detect then crop ticks in submitted images with a state-of-the-art object detection algorithm, (ii) enhance the image using super-resolution with GANs, and (iii) classify ticks in four datasets grouping user-submitted tick images in categories of decreasing taxonomic resolution (17, 12, 6 and 3 classes). Two GANs were tested for classification: RealESRGAN, which uses convolutions, and SwinIR, a more advanced GANs that uses transformers. For the classification task, different ResNets (32-50-101) were tried against a transformer architecture ViT (Vision Transformer).

## 2. Materials and Methods

### 2.1. General Methodology

**Figure 2:**
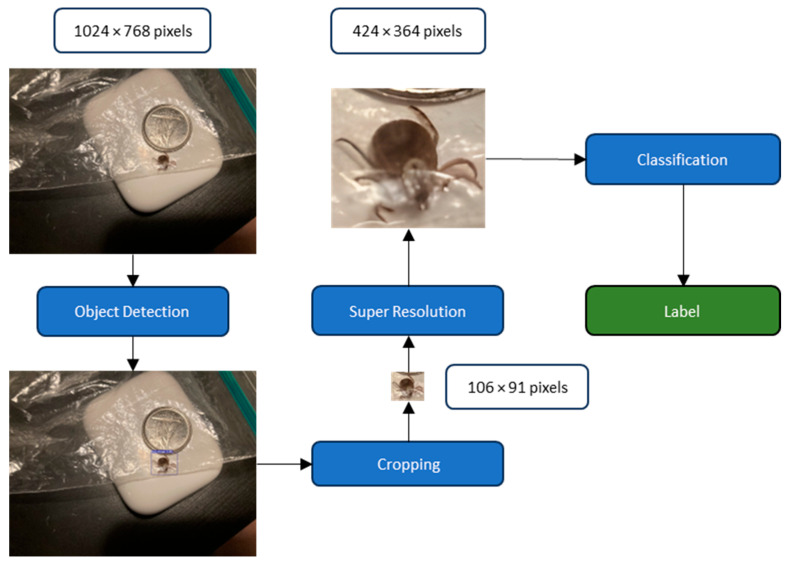
Flowchart of the proposed methodology. An object detection model first locates the tick in the picture and the image is then cropped around the tick. A super resolution GANs then enhances the cropped image before a classification model assigns an identification label to the tick in the image.

The general methodology can be summarized as follows. An object detection model first locates the tick in the picture, and the image is then cropped around the tick. A super-resolution GAN subsequently enhances the cropped image before a classification model assigns an identification label to the tick (Figure 2).

### 2.2. Image Collection and Initial Class Attribution

We selected 1412 images submitted to the eTick platform and identified by trained personnel for our master dataset. Data can be downloaded at https://github.com/ettelephonne/Ticks-classification-project (accessed on 25 July 2025). These images were selected to represent the most common tick species, life stages (males, females + nymphs when highly similar to females) and viewing angles (dorsal and ventral) submitted onto the eTick platform. Given that dorsal views are necessary for most tick identifications, these were prioritized when selecting eligible species-stage combinations. Only species-stages combinations with at least 50 submissions were included in the dataset to ensure that at least 50 pictures would be available for training. No more than three images from the same specimen were included in the dataset. Ventral views were assigned to a species based on their association with at least another dorsal image from the same specimen in the original eTick submission. Given that ventral views are common but not systematically submitted by eTick users, these were included in the analyses even when fewer than 50 images were available for a given species-stage-viewing angle combination to determine if AI could identify these tick species from ventral views alone, and should this fail, at least separate these ventral views from dorsal ones.

Each image was assigned to one of 17 original classes (Table 1). The 17-class dataset also includes the Bad Image class, which was created to encompass all images in the dataset that were too low in quality to qualify as a representative dorsal or ventral view of the species (according to our trained personnel). Following initial model training, we concluded that our original definition for “Bad Image’’ was too conservative as the model was able to classify some of these to the correct class (species/stage/viewing angle combination). We therefore underwent an iterative process where we used the algorithm’s incorrect predictions to re-evaluate which class images belonged to either Bad Image or their confirmed class based on eTick data in hopes of improving the algorithm’s performance. The final sample size for each class following that iterative process can be found in Table 1. The uneven distribution of images for each class is explained by the uneven representation of taxa, sex, and specimen viewing angle submitted on the eTick platform where *Dermacentor* sp. (a group of 4 nearly identical species dominated by *D. variabilis* and assigned to a single taxon) and *Ixodes scapularis* (the blacklegged tick) represent approximately 62% and 32% of total submissions, respectively. Female ticks are more commonly submitted than males, especially for *Ixodes species*, and ventral views of specimens are not systematically submitted by eTick users.

### 2.3. Detection and Cropping

YOLO (You Only Look Once) is a one-stage detector that directly predicts object categories and bounding box coordinates from full images in a single forward pass of the network. The seventh iteration of YOLO [25] was chosen as it performed very well on different benchmarks [26] and is easy to fine-tune. This enables the precise localization of ticks within the images. YOLOv7’s ability to rapidly and accurately identify and draw bounding boxes around ticks is crucial, especially given the small size of the ticks and the often-cluttered backgrounds of the submitted images.

The data annotation was facilitated using the online tool MakeSense.ai, which provides an intuitive interface for image annotation. The annotator first uploaded the images to the platform, and then, using the tool’s functionalities, drew rectangles around each tick. These rectangles were positioned to encompass the entire tick but excluded as much of the non-relevant background as possible. Each bounding box was then labeled as one of 17 classes (Table 1).

We fine-tuned a YOLOv7 object detection model to locate ticks in user-submitted images, starting from a model pre-trained on the MSCOCO dataset [27]. The training process was tracked using Weights & Biases (wandb) [28] for continuous monitoring of performance metrics. Following the YOLOv7 authors’ recommendations, all images were resized to 640 × 640 pixels (https://github.com/WongKinYiu/yolov7 (accessed on 3 July 2025)), and the dataset was split into approximately 80% training and 20% validation images. The batch size was set to 24 to fully utilize the available GPU memory (16 GB). Once a tick was detected, the coordinates of the bounding box were utilized to crop the image around the tick. To ensure that no part of the tick was inadvertently cropped out, a buffer of 10% of the bounding box size was added to all sides of the crop, providing a margin that safeguards the integrity of the tick’s image.

### 2.4. Resolution Enhancement

The cropping process can result in images that are smaller than required for subsequent processing steps. Since Vision Transformer (ViT), one of the models chosen for the final classification step (see below), necessitates input images of size 224 × 224 pixels, any cropped images of smaller dimension will need to undergo a super-resolution process.

Super-resolution GANs leverage the adversarial training framework, where two networks, a generator and a discriminator, are trained simultaneously. The generator aims to produce high-resolution images that are indistinguishable from real high-resolution samples, while the discriminator’s objective is to differentiate between the generator’s outputs and authentic high-resolution images. This competition drives the improvement of the generator’s output quality. Several architectures have been pivotal in advancing the field such as those presented in this work: RealERSGAN and SwinIR.

Real-ESRGAN aims at enhancing real-world images through practical restoration tasks. It extends ESRGAN by focusing on more challenging real-world scenarios where images may suffer from various degradation patterns, such as noise, blur, and compression artifacts. Real-ESRGAN introduces several key improvements. It is trained with a more diverse dataset that includes synthetic and real-world degradations, making it robust against a wide range of image quality issues. Modifications in the network architecture ensure better handling of diverse degradation types. The incorporation of new loss functions that better capture the characteristics of real-world degradations, leading to more natural and plausible super-resolution results [29].

SwinIR represents a shift towards using transformer-based models for image super-resolution, utilizing the Swin Transformer as its backbone. The Swin Transformer is a hierarchical transformer whose representation is computed with shifted windows, enabling efficient modeling of long-range dependencies. SwinIR’s use of the Swin Transformer allows it to flexibly model both local and global context, which is beneficial for restoring fine details in super-resolution tasks. On various benchmark datasets, SwinIR has demonstrated superior performance, particularly in recovering fine textures and details, surpassing previous GAN-based and CNN (convoluted neural networks)-based models [30].

One of our aims was to upscale the cropped images to a higher resolution that maintains the quality and integrity of the tick features necessary for accurate classification. This approach is not intended to counteract the initial resampling to a 640 × 640 resolution but rather to refine the quality of the smaller cropped images resulting from the localization process. A conventional upscaling method, such as bilinear or bicubic interpolation, while simpler, would be likely to introduce blurring and fail to restore the high-frequency details that are crucial for the subsequent classification task. By employing super-resolution, we enhanced the cropped images beyond mere upscaling. The super-resolution process aims to reconstruct a high-resolution image from the low-resolution crop by inferring and reintroducing fine details that were lost, resulting in a higher-quality image. This provided our classification models with more detailed and nuanced representations of the ticks, creating better conditions for the accurate classification of the tick image.

In our methodology, we deliberately chose not to follow the advice suggesting the use of a higher resolution during fine-tuning than during the pre-training phase of the Vision Transformer (ViT) [31,32] The rationale behind our decision was twofold. Firstly, the tick images we encounter are often very small. Upscaling these images to a resolution much higher than 224 × 224 could amplify noise or artifacts, which may hinder the model’s ability to learn meaningful representations. Secondly, the ViT model we employed was optimized and pre-trained specifically on 224 × 224 images. This pre-training tailors the model’s parameters, including its positional embeddings, to this resolution. By maintaining the 224 × 224 resolution, we leveraged the full strength of the pre-training, ensuring that the fine-tuning process aligned with the model’s initial conditions. This approach mitigated the risk of introducing misalignments between the pre-trained positional embeddings and the upscaled images, which could occur if the embeddings were interpolated for a higher resolution. The same reasoning applies to the ResNet model.

Since no actual ground truth is available for our specific task, and creating a task-specific training dataset is beyond the scope of our work, we decided to visually evaluate the results of the two architectures, RealESRGAN and SwinIR. Although visual assessment is inherently subjective and lacks the precision of quantitative metrics, it offers a pragmatic solution in the absence of ground truth. This approach allows experts in the field of tick identification to use their knowledge to make informed judgments about the quality of the reconstructions. While not ideal, this evaluation method provides a basis for assessing the performance of the super-resolution models and guides our decision-making process in selecting the architecture that produces the most realistic and detailed images.

### 2.5. Image Classification

Image classification is a foundational task in the field of computer vision, where the goal is to categorize the entire image into one of the predefined classes. Deep learning has revolutionized image classification, first with CNNs and, more recently, with transformer models achieving state-of-the-art performance.

Resolution Network (ResNet) is a milestone in CNN architecture as it addresses the problem of training very deep networks through the introduction of “residual blocks” with skip connections. These connections allow gradients to flow through the network more effectively, enabling the training of networks that are much deeper than was previously feasible. Each block has a shortcut connection that skips one or more layers, which helps in mitigating the vanishing gradient problem. ResNet models come in various depths, including ResNet-50, ResNet-101, and ResNet-152, offering flexibility and scalability. By enabling the training of deeper networks, ResNet significantly improved performance on image classification tasks. ResNet’s introduction [33] marked a significant advancement in deep learning, making it possible to train networks with hundreds of layers effectively. It has since become a foundational model for a wide range of computer vision tasks beyond classification, including object detection and segmentation.

Vision Transformer (ViT) adapts the transformer architecture for image classification tasks [31]. ViT treats an image as a sequence of fixed-size patches, processes these patches through a series of transformer blocks, and applies self-attention across patches to model their relationships. ViT divides the image into patches and linearly embeds each of them, akin to tokens in NLP (Natural Language Processing) tasks. The self-attention mechanism allows the model to consider the entire image at once, capturing global dependencies that CNNs might miss. ViT’s performance improves significantly with scale, benefiting from larger datasets and models, in contrast to the diminishing returns observed in CNNs beyond a certain depth and demonstrated that transformers could achieve state-of-the-art results in image classification, challenging the dominance of CNNs. Its success has spurred further research into transformer-based models for a broad range of vision tasks, leading to developments like Swin Transformer and others that build on the ViT framework.

In the present work, we tested and compared two different architectures, namely ResNet and ViT, to evaluate which one would perform best at identifying ticks from public-generated images. Given the different ResNet models available, such as ResNet-32, ResNet-50, and ResNet-101 used by [19], we started by determining which one of these would perform best for this task. As ResNet-50 achieved the best results during our preliminary tests, this architecture was retained to be compared with ViT, the state of the art of image classification.

For the final classification tasks, three datasets of decreasing taxonomic resolution were tested (namely the 12, 6, and 3 classes datasets in Table 2). Although the original dataset included 17 classes, preliminary tests with ResNet and ViT quickly confirmed that the complexity of this dataset was too great to achieve satisfactory classification, particularly when considering the lower sample sizes of certain classes. As such, we focused our initial classification task on the 12-class dataset. Following each round of model outputs some classes were merged to improve model performance in terms of classification (Table 2). These new classes were based on groupings that would be useful if implemented in the eTick workflow.

The empirical evaluation of ResNet-50 and Vision Transformer (ViT) models was conducted using a validation set, constituting 10% of the whole dataset. This dataset comprised images precisely cropped around ticks as detailed previously. Both ResNet-50 and ViT were leveraged from their ImageNet-pretrained states, a strategic move to capitalize on the vast, generalized feature representations learned from a diverse and extensive corpus of visual data. The training regimen for both architectures was harmonized, employing a starting learning rate of 1 × 10^−4^, a batch size of 128, and Lion as the optimizer [34]. The learning rate was subjected to an adaptive decay mechanism, diminishing by a factor of 0.2 for every two epochs devoid of performance gains, with the training culminating after four successive adjustments. This approach underscores a commitment to precision and computational efficiency, terminating the learning process upon saturation to avoid overfitting and resource exhaustion.

### 2.6. Metric Choice

Some of the tick species included in our dataset are of significant medical relevance, prompting us to select the F1 score, defined as follows, as our primary evaluation metric for this study.

F1-score = 2 × (Precision × Recall)/(Precision + Recall)

The F1-score is particularly suited for evaluating models in contexts where a balance between precision and recall is crucial. Precision measures the correctness of positive identifications made by the model, an important metric when the cost of misidentification can be high, while recall assesses the model’s ability to correctly identify all instances of a given tick species in the dataset. The F1-score combines these two metrics into a single value, providing a more comprehensive assessment of the model’s performance.

In our study we used the macro-averaged F1-score to handle the multiclass nature of the data. This approach computes the F1-score for each class independently, treating each as “one versus all,” and then calculates the unweighted average across all classes. By doing so, the metric ensures that all classes are equally represented, regardless of their frequency in the dataset.

## 3. Results and Discussion

### 3.1. Object Detection

**Figure 3:**
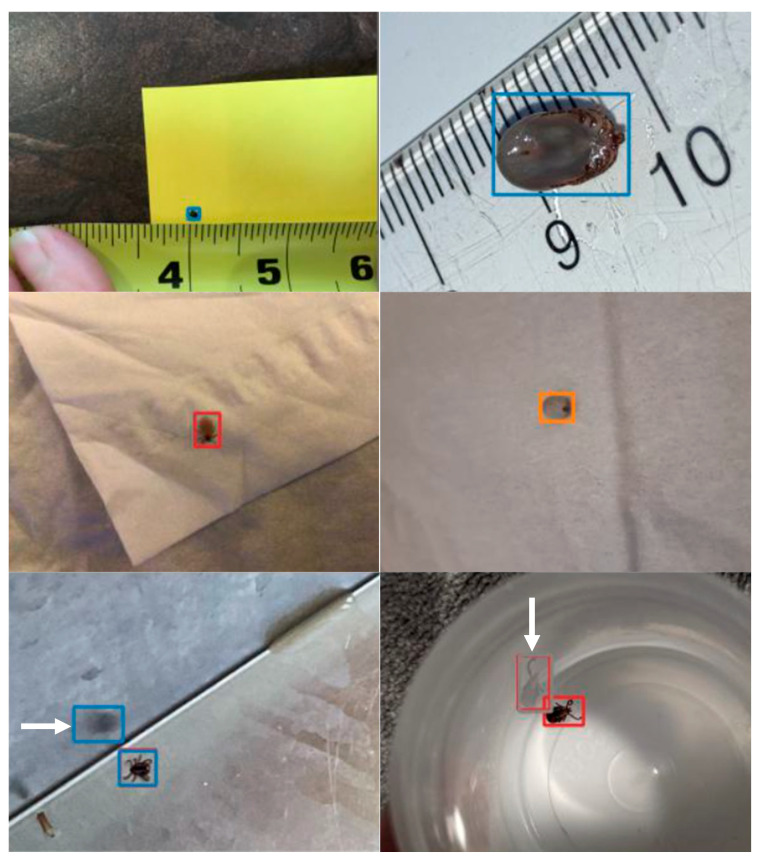
Examples of bounding boxes predicted on the validation set. The ticks are correctly detected but some reflections (identified by white arrows) were detected as ticks by the model.

Initially, we attempted to train the model using all 17 classes defined in our dataset (Table 1), which include fine distinctions between species, and dorsal/ventral views. However, this level of taxonomic granularity proved unrealistic for an object detection task, given the relatively small size of the tick in the 640 × 640 resized images. The downscaling process caused the loss of many of the fine morphological details required to distinguish between species and stages, making such a classification problem unfeasible at the detection stage. As a result, the model struggled to classify the ticks correctly among the 17 classes, achieving a low macro-averaged F1-score of only 59.2%. To overcome this limitation, we reframed the task: rather than using object detection to classify the tick species, we simplified the detection step to a single “tick” class, independent of species or stage. This approach allowed the model to focus solely on localizing the tick in the image without having to resolve fine taxonomic differences lost during image resizing. With this simplification, the detection performance drastically improved, achieving a macro-averaged F1-score of 99.5%, demonstrating that YOLOv7 is highly effective at detecting the presence of a tick, even if species-level classification requires a separate, higher-resolution step. Interestingly, several instances of false positives for detection involved reflections of ticks being detected as ticks (Figure 3).

Precise localization is essential in practical scenarios because it allows for the isolation of the relevant section of the image for further analysis. This consistent detection performance, even in the absence of species identification, demonstrates that the model maintains robust detection capabilities. Such reliable detection is critical for subsequent stages of image processing, where the localized tick will undergo more detailed, higher-resolution analysis. The contrast between the model’s strength in detecting ticks and its struggle with species identification underscores the importance of managing trade-offs between detection accuracy and classification tasks.

### 3.2. Resolution Enhancement

**Figure 4:**
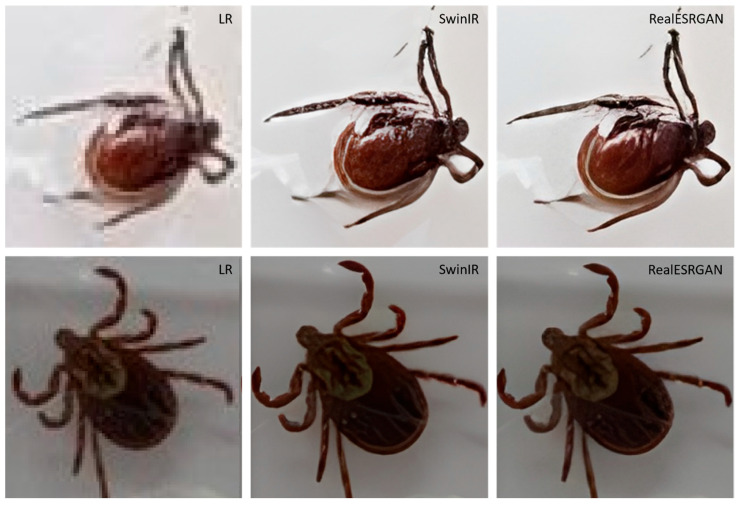
Comparison of cropped images at original low resolution (LR) alongside the upscaled resolution images produce by SwinIR and RealERSGAN for a male *Ixodes scapularis* (**top**) and a female *Dermacentor* spp. (**bottom**).

The comparative analysis of image super-resolution results obtained from RealESRGAN and SwinIR revealed a remarkably close performance between the two architectures. Both were successful in upscaling by a factor of 4 and effectively removing compression artifacts from the images. A subtle edge was observed in the reconstruction of certain details with RealESRGAN, which contributed to our decision to favor this architecture (Figure 4).

We acknowledge that a more exhaustive investigation into the various parameters and settings of SwinIR might have swayed our evaluation towards it. There is a possibility that fine-tuning SwinIR’s parameters could have exploited its potential further and yielded even better results. However, such an extensive parameter search would have extended beyond the primary focus of this project.

Similarly, the idea of fine-tuning these models on tick-specific images was not pursued. The primary reason for this being the high quality of the results obtained from the pre-trained models and the lack of a specialized training dataset closely matching our target domain. The models we assessed were trained on the DIV2K dataset, a benchmark collection in the super-resolution community known for its high-resolution and diverse set of images. Without a similarly comprehensive and relevant training dataset for ticks, the likelihood of significantly improving the models’ performances through fine-tuning was uncertain.

### 3.3. Classification

**Figure 5:**
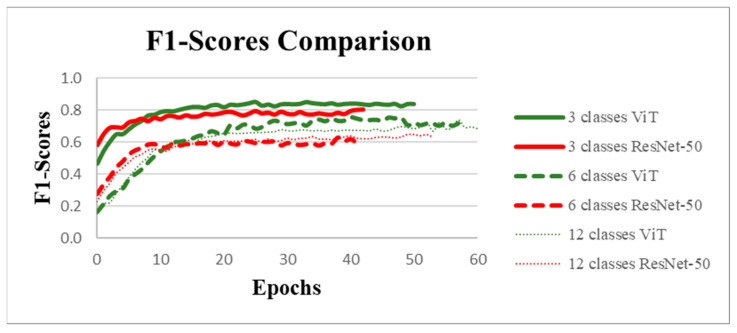
Comparison of F1-score performance between Vision Transformer (ViT) and ResNet-50 models for classification tasks with 3, 6, and 12 classes. The curves represent the evolution of F1-scores over training epochs. The results indicate that ViT outperforms ResNet-50.

We compared the F1-scores of ResNet-50 to one of the state-of-the-art architectures ViT for each of the three datasets (Figure 5). For each dataset (12, 6, and 3 classes), the Vision Transformer (Vit) achieved better results, suggesting that ViT’s attention mechanisms and ability to capture long-range dependencies provide an advantage over ResNet-50’s convolutional layers. Another important trend is the relationship between the number of classes and the performance. As the number of classes decreased, the F1-scores improved. This was particularly noticeable with the 3-class scenario, where both architectures performed significantly better than with 6 or 12 classes. Starting at 6 classes, the complexity became too high for ResNet-50, causing performances to collapse at both 6 and 12 classes. We therefore concluded that ViT is a better choice for this classification task, especially when the number of classes was higher. Its superior ability to extract features and generalize across classes suggests that it may be more suitable for complex tasks where traditional convolution-based architectures like ResNet-50 might fall short.

**Figure 6:**
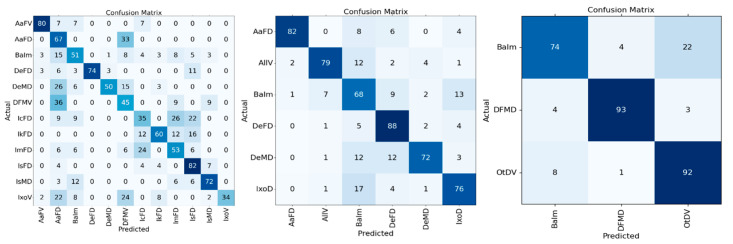
Comparison of confusion matrices for tick classification models for the 12, 6 and 3 classes datasets with recall values normalized by the number of occurrences to facilitate readability (percentages). All values are rounded to the nearest integer for clarity.

All of our datasets exhibited class imbalance, which inherently complicates model training by skewing the learning bias towards more populous classes. The impact of this imbalance on F1-scores, particularly for models burdened with a higher number of classes, remains a crucial area of inquiry. The analysis of confusion matrices at different levels of class granularity (Figure 6) allowed us to further investigate class-level performance and performance/granularity trade-offs. The 12-class matrix yielded an average correct classification of 59%, with some classes (AaFV, DeFD, IsFD, IsMD) predicted with over 70% recall while others (*Dermacentor* V, IcFD, IxoV) showed less than 50% recall and high inter-class confusion. Regrouping into 6 classes increased the overall recall by 17.5% points to 77.5%, with five out of six classes exceeding 70% recall. BaIm (Bad image) had the lowest recall with 68%. Further regrouping into 3 classes achieved an 86.3% recall (an additional 8.8 point gain) and over 90% recall for DFMD and OtDV. The lowest score was again observed in the BaIm class (74%). Unlike all others, “Bad Image” is a class with a broad subjective definition that is open to interpretation. It is characterized by blurring, poor contrast, suboptimal angles, and/or incomplete tick representation often due to obstruction by other components of the image, leading to high rates of misclassification.

Although recall is an important metric for the evaluation of model performance, particular attention must be paid to classification errors when it comes to ticks, given that medical relevance is taxon-specific. Focusing on the best-performing model (3-class model), the most important identification error was that a single image (0.7%) of OtDV (other species, a class that includes medically relevant species like *Ixodes scapularis* and *Amblyomma americanum*) and ~5% of BaIm (bad images which could be from any species) were classified as *Dermacentor* spp. (DFMD), a group of lesser medical relevance in Canada. Such identification errors could therefore result in a submitter receiving incorrect information and advice following a tick bite. Although ~1% and 5% remain low, mitigation procedures such as confidence thresholds to flag poorly supported class assignments could be implemented to reduce the risk of misclassification for certain groups.

Other errors of less consequences in the 3-class model include 22% of bad images (BaIm) and 3% of *Dermacentor* sp. (DFMD) being misclassified as OtDV (i.e., other species), and 4% of DFMD and 8% of OtDV being misclassified as bad images. Given that these categories (OtDV and BaIm) would still require trained personnel for further assessment (species identification or rejection for quality reasons), such classification errors would be corrected before results are communicated to the submitter.

## 4. Conclusions

Based on our findings, we propose the following framework for building an AI-based classification tool from public-generated (i.e., unstandardized) images. First, a representative sample of images must be used to train programs such as YOLO to detect the desired objects accurately (tools such as MakeSense.ai can aid in this step). Second, to decrease the effects of small relative size and resulting resolution issues after object detection and cropping, programs like RealESRGAN (SwinIR may also work) can be used to upscale images to the appropriate format (based on the chosen classification model requirements) and optimize resulting image resolution. Third, a ViT model can be trained using a representative sample of images (being careful of inherent biases that can result from sample size variations between classes). At this step, and depending on the task, adjustments to the class structure of the data may be needed to optimize model performance. Finally, once the framework is in place, performance should be evaluated to assess whether the model performs within an acceptable error margin for the task and determine the implications of different classification errors to decide if/how the framework can be implemented within a project. The implementation of this framework in other initiatives should allow for faster identification of target taxa (including pest species) and save considerable resources which could be re-allocated to service development and improvement. This will undoubtfully benefit both the project’s managers and participants. Of course, given the rapid pace at which the field of artificial intelligence and image recognition is growing, the programs/architectures discussed above may be outperformed by others, but the general framework should still be relevant in years to come.

Regarding the practical applications of our framework to the eTick platform, it will be implemented as a back end pre-filtering tool (i.e., not visible to the public), allowing new submissions to be pre-filtered as “*Dermacentor*”, “Other” or “Bad image” prior to being processed by identification personnel. Considering that in Canada the overall medical relevance of *Dermacentor* ticks is generally considered lower than that of other taxa like *I. scapularis* or the adventitious *A. americanum*, and that the requirement to correctly identify ticks of medical relevance is high, this will allow identification personnel to prioritize the review of “Other” submissions, a diverse and challenging class that includes the most medically relevant tick species. *Dermacentor* submissions will be processed last and more efficiently considering personnel will only need to confirm the identification and verify the accuracy of the submission’s information. Considering that *Dermacentor* makes up more than 60% of online submissions to the eTick platform, this new framework, paired with the cropping and resolution improvements, should reduce and accelerate the workload of identification personnel. This could save the project hundreds of hours and associated salary each year, without sacrificing identification accuracy. Of course, we aim to further train and improve our models over time, hoping to increase the number of classes and potentially implement the framework in a way that provides feedback to submitters almost instantaneously. However, considering the framework remains to be tested on the actual platform, and considering the potential consequences of misclassifying a more medically relevant species like *I. scapularis* tick as *Dermacentor*, we currently favor a cautious back end deployment approach.

Further optimization of our proposed framework could also be implemented. For instance, although model accuracy and recall rates are primary concerns when developing AI classification models, their heavy computational demands must also be considered, especially in the context of mobile platforms. Fortunately, this can be mitigated by deploying these models on server-based infrastructure, allowing the servers to do the heavy lifting, and relieving the computational constraints for the mobile platform. This is how we intend to implement our model on the eTick platform, and by extension, the eTick mobile applications. Building on the results presented here, we are currently developing a two-step enhancement of the classification pipeline. First, we aim to integrate a dedicated model trained to assess image quality, capable of distinguishing between “acceptable quality” and “low quality” submissions. This model would operate upstream of the classification step and allow for near real-time feedback to the user, prompting them to retake photographs if necessary. Second, for submissions with images deemed of sufficient quality, we plan to implement a multi-view classification approach, where all available views (e.g., dorsal and ventral) of the same specimen are aggregated into a single input object. This strategy is expected to increase the probability of capturing a diagnostically useful view and to mitigate the effects of suboptimal individual images on overall classification performance. While our new framework was developed to streamline and accelerate the identification and messaging processes of the eTick platform in Canada we expect that some components will be transferable to other image-based initiatives worldwide.

## Figures and Tables

**Table 1:** Image classes, four letter class code, and number of images included for each class of the dataset.

Image Class	4 Letter Code	Number of Images
Bad Image	BaIm	315
*Dermacentor* * Female Dorsal	DeFD	138
*Dermacentor* Female Ventral	DeFV	24
*Dermacentor* Male Dorsal	DeMD	133
*Dermacentor* Male Ventral	DeMV	20
*Ixodes scapularis* Female ** Dorsal	IsFD	111
*Ixodes scapularis* Female Ventral	IsFV	42
*Ixodes scapularis* Male Dorsal	IsMD	127
*Ixodes scapularis* Male Ventral	IsMV	17
*Ixodes cookei* Female ** Dorsal	IcFD	100
*Ixodes cookei* Female Ventral	IcFV	60
*Ixodes kingi* Female Dorsal	IkFD	101
*Ixodes kingi* Female Ventral	IkFV	58
*Ixodes marxi* Female Dorsal	ImFD	67
*Ixodes marxi* Female Ventral	ImFV	31
*Amblyomma americanum* Female Dorsal	AaFD	59
*Amblyomma americanum* Female Ventral	AaFV	9

* The four highly similar *Dermacentor* species found in Canada are grouped in a single unit. ** Some nymphs may also be included in these classes as they are highly similar to females.

**Table 2:** Classes included in each dataset; F: Female, M: Male, D: Dorsal, V: Ventral. Merged classes include all classes in matching rows from the previous columns.

17 Classes	12 Classes	6 Classes	3 Classes
Bad Image	Bad Image	Bad Image	Bad Image
*Dermacentor* FD	*Dermacentor* FD	*Dermacentor* FD	*Dermacentor* D
*Dermacentor* MD	*Dermacentor* MD	*Dermacentor* MD	
*A. americanum* FD	*A. americanum* FD	*A. americanum* FD	Others DV
*I. scapularis* FD	*I. scapularis* FD	*Ixodes* D	
*I. scapularis* MD	*I. scapularis* MD		
*I. cookei* FD	*I. cookei* FD		
*I. kingi* FD	*I. kingi* FD		
*I. marxi* FD	*I. marxi* FD		
*A. americanum* FV	*A. americanum* FV	All V	
*Dermacentor* FV	*Dermacentor* V		
*Dermacentor* MV			
*I. scapularis* FV	*Ixodes* V		
*I. scapularis* MV			
*I. cookei* FV			
*I. kingi* FV			
*I. marxi* FV			

## Data Availability

https://github.com/ettelephonne/Ticks-classification-project (accessed on 25 July 2025).
